# Geostatistical exploration of dataset assessing the heavy metal contamination in Ewekoro limestone, Southwestern Nigeria

**DOI:** 10.1016/j.dib.2017.07.041

**Published:** 2017-07-21

**Authors:** Kehinde D. Oyeyemi, Ahzegbobor P. Aizebeokhai, Hilary I. Okagbue

**Affiliations:** a Department of Physics, Covenant University, Ota, Nigeria; b Department of Mathematics, Covenant University, Ota, Nigeria

**Keywords:** Ewekoro limestone, MPAS, Heavy-metal contamination, Cluster analysis, Pearson correlation coefficient, Kendall tau, Spearman rho

## Abstract

The dataset for this article contains geostatistical analysis of heavy metals contamination from limestone samples collected from Ewekoro Formation in the eastern Dahomey basin, Ogun State Nigeria. The samples were manually collected and analysed using Microwave Plasma Atomic Absorption Spectrometer (MPAS). Analysis of the twenty different samples showed different levels of heavy metals concentration. The analysed nine elements are Arsenic, Mercury, Cadmium, Cobalt, Chromium, Nickel, Lead, Vanadium and Zinc. Descriptive statistics was used to explore the heavy metal concentrations individually. Pearson, Kendall tau and Spearman rho correlation coefficients was used to establish the relationships among the elements and the analysis of variance showed that there is a significant difference in the mean distribution of the heavy metals concentration within and between the groups of the 20 samples analysed. The dataset can provide insights into the health implications of the contaminants especially when the mean concentration levels of the heavy metals are compared with recommended regulatory limit concentration.

**Specification Table**

**Table:** 

Subject area	*Earth, Environment and Planetary science*
More specific subject area	*Environmental Science*
Type of data	*Table and Figure*
How data was acquired	*Microwave Plasma Atomic Absorption Spectrometer.*
Data format	*Raw, Analysed*
Experimental factors	*The collected samples went through a drying process in bid to make it air free, it was grounded and sieved again. 2 g of the samples was placed in a beaker, 2.5 ml of concentrated HNO_3_ and 10 ml of concentrated HCl was added to them and then covered with a watch glass. The beaker was then placed on a hot plate for 15 min to heat. The digestate from the heated sample was filtered using a Whatman No. 41 filter paper into a 100 ml volumetric flask. The digestate was later diluted to a volume of 100 ml and then analysed using a Microwave Atomic Absorption Spectrometer.*
Experimental features	*Determination of Arsenic (As), Mercury (Hg), Cadmium (Cd), Cobalt (Co), Chromium (Cr), Nickel (Ni), Lead (Pb), Vanadium (V) and Zinc (Zn) elemental contaminations.*
Data source location	*Ewekoro limestone formation in the eastern Dahomey basin, Southwestern Nigeria*
Data accessibility	*All the data are in this data article*.

**Value of the data**

• The data could be used to determine the level of heavy metal contamination in limestone formations.
• The methods can be replicated to other rock formations. For example to other two key lithostratigraphic units of the eastern Dahomey Basin namely; Abeokuta and Akinbo Formations.
• For educational purposes, environmental pollution studies especially in the study of heavy metals in fossiliferous limestone. Similar data articles can be found in [1], [2], [3], [4], [5], [6], [7], [8], [9], [10], [11], [12], [13].
• Findings can be extended to other metal or non-metal elements not considered in this article.
• The dataset can provide insights on the health implications of the contaminants on the groundwater especially when the mean concentration levels of the heavy metals are compared with recommended regulatory limit concentration.

## 1 Data

The data contains geostatistical analysis of twenty (20) samples of limestone obtained from the Ewekoro limestone Formation in the eastern Dahomey basin, Southwestern Nigeria. The samples were purified and analysed for heavy metal concentrations using the MPAS. The heavy metals detected from the samples are Arsenic (As), Mercury (Hg), Cadmium (Cd), Cobalt (Co), Chromium (Cr), Nickel (Ni), Lead (Pb), Vanadium (V) and Zinc (Zn). The detailed composition is shown in Table 1. The presence of these heavy metals causes contamination. The descriptive statistics is shown in Table 3. Further analysis was conducted to deepen our understanding on the statistical relationships of the samples. The analysis can be replicated on other limestone Formations and the mean heavy metal concentrations can be compared with the recommended limits.

**Table 1:** The heavy metal concentrations of the 20 samples in (mg/kg).

Sample	Arsenic	Mercury	Cadmium	Cobalt	Chromium	Nickel	Lead	Vanadium	Zinc
1	0.2028	0.3779	0.1319	0.01	0.0895	0.227	1.1785	0.1361	0.3111
2	0.6672	0.0511	1.4303	0.0138	0.7368	0.2939	1.347	0.3697	0.3659
3	0.4671	0.3051	0.0209	0.0039	0.05	0.3021	0.1071	0.162	0.0272
4	0.0867	0.2282	0.1145	0.0115	0.0554	0.3652	0.0522	0.1169	0.2425
5	0.8505	0.1407	1.1572	0.0219	0.1331	0.5209	1.9295	0.3019	2.1426
6	0.0505	0.1872	0.5401	0.0297	0.1235	0.1663	0.3739	0.0364	0.0601
7	1.1031	0.108	1.8967	0.0342	0.7252	0.4089	0.6685	0.9679	0.4062
8	0.0612	0.083	0.2675	0.0342	0.0325	0.2437	0.0769	0.0903	0.1403
9	0.0312	0.1156	0.33	0.0352	0.0141	0.2406	0.0778	0.0145	0.0603
10	0.2578	0.0405	0.8362	0.0333	0.2556	0.3455	0.8067	0.1756	2.5526
11	0.002	0.1071	0.0143	0.0112	0.0044	0.345	0.0558	0.0887	0.0263
12	0.0693	0.0466	0.5039	0.0496	0.1254	0.2049	0.0836	0.22	0.4097
13	0.0432	0.087	0.1791	0.0365	0.0016	0.2997	0.0124	0.1841	0.7204
14	0.3571	0.0226	0.5523	0.0274	0.2706	0.297	0.214	0.1234	0.8286
15	0.5042	0.1524	0.0453	0.0234	0.0714	0.4165	0.1137	0.2199	0.2122
16	0.1018	0.0016	0.9061	0.0235	0.0869	0.1454	0.4061	0.1072	0.0591
17	0.7089	0.1151	1.3779	0.0249	1.1412	0.2282	0.433	1.0141	0.5636
18	0.0494	0.0723	0.306	0.0425	0.1115	0.2391	0.0233	0.1498	0.3992
19	0.0253	0.0537	0.1946	0.052	0.0122	0.3187	0.0195	0.054	0.7042
20	0.1717	0.0157	1.0858	0.0246	0.4546	0.3076	0.2976	0.3291	0.4758

## 2 Experimental design, methods and materials

Several data analysis has been carried out on the physical, geological and geophysical characteristics of the Ewekoro limestone formation in the eastern Dahomey basin, Southwestern Nigeria. Some of the works include: factors causing differentials in the shear velocities, Lithotype representation by Nuclear Magnetic Resonance (NMR) and blastability properties, investigation of allochemical and orthochemical components of the limestone formation, ground vibration and noise generated during blasting, gas generating potential and prospects, estimation of thermal conductivity, assessment of reservoir potential, isotopic elements composition and diagenesis. Others include: Organic geochemical analysis and appraisal, elemental analysis, distribution of tree oxides and groundwater composition [14], [15], [16], [17], [18], [19], [20], [21], [22], [23], [24], [25], [26], [27], [28].

### 2.1 Study area

**Fig. 1:**
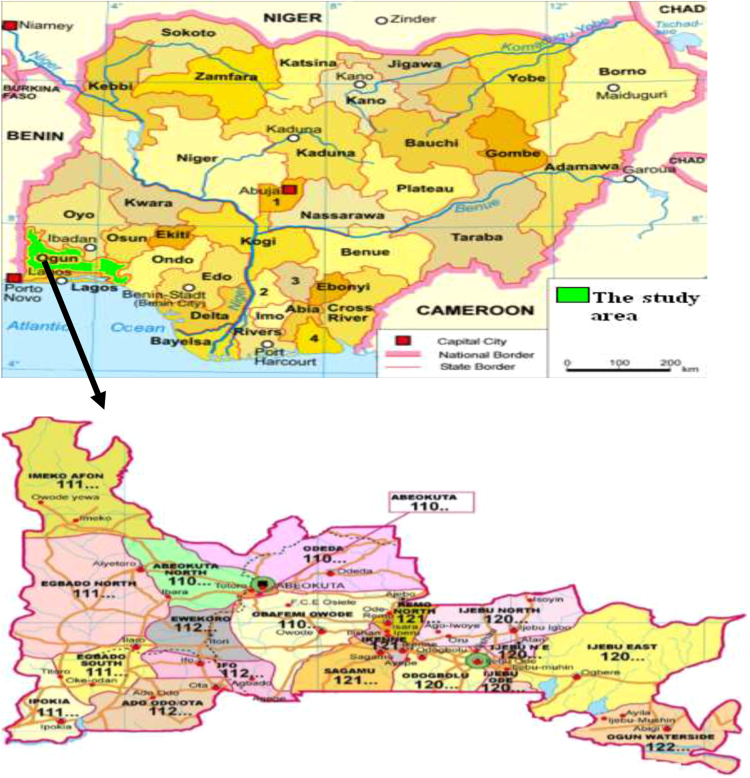
Map of Nigeria showing the location of study. Source: Google Maps [21].

The study area lies between longitude 3°05′E to 3°15′E and latitudes 6°40′N to 6°55′N and situated within the Ewekoro local government area, Ogun state, southwestern part of Nigeria. It is bounded by Lagos state to the South, Osun state and Oyo state to the North, the republic of Benin to the West and Ondo state to the East. It. The map indicating the study area is shown in Fig. 1.

The geology of the study area is that of Eastern Dahomey Basin, a combination of inland/coastal/offshore basin that stretches from south-eastern Ghana through Togo and the Republic of Benin to south-western Nigeria. It is separated from the Niger Delta by a subsurface basement high referred to as the Okitipupa Ridge which marks the continental extension of the fracture zone. Its offshore extent is poorly defined. Sediments deposition within the Dahomey Basin follow east-west trend, and the stratigraphy consists of six geological formations comprising Abeokuta, Ewekoro, Akinbo, Oshosun, Ilaro and Benin Formations. The Cretaceous Abeokuta Formation is a non-fossiliferous basal sequence resting on the Precambrian basement. The overlying Ewekoro Formation is a shallow marine limestone; Paleocene in age and belongs to the Tertiary-formed Paleocene and Eocene sedimentary formations. The formation is composed of non-crystalline and highly non-fossiliferous limestone. It is also composed of thinly laminated, fissile and non-fossiliferous shale. Ewekoro Formation is overlain by a shale-dominated Akinbo Formation that is of Late Paleocene-Early Eocene.

### 2.2 Sample collections

Limestone samples were collected from some rock outcrops within Ewekoro local government area, twenty (20) samples in total with their GPS coordinates recorded in Table 2. These samples were then filtered using a sieve in order to remove pebbles and other irrelevant materials which may affect the result during the analysis. These samples were then packaged into neat polyethylene bags and labelled orderly for identification.

**Table 2:** Twenty collected samples and their GPS coordinates.

Sample number	Easting	Northing
Sample 1	3.69561	6.51619
Sample 2	3.69554	6.5162
Sample 3	3.69569	6.51621
Sample 4	3.68957	6.51613
Sample 5	3.68955	6.52616
Sample 6	3.70195	6.52615
Sample 7	3.70201	6.51708
Sample 8	3.70206	6.51703
Sample 9	3.69612	6.51712
Sample 10	3.70213	6.51591
Sample 11	3.70203	6.51707
Sample 12	3.69606	6.51712
Sample 13	3.69617	6.51594
Sample 14	3.6959	6.51589
Sample 15	3.70105	6.5157
Sample 16	3.70105	6.51682
Sample 17	3.70112	6.51694
Sample 18	3.70128	6.51692
Sample 19	3.70168	6.51711
Sample 20	3.70136	6.51701

### 2.3 Samples preparation

The samples went through a drying process in bid to make it air free, it was grounded and sieved again. 2 g of the sample was placed in a beaker; 2.5 ml of concentrated HNO_3_ and 10 ml of concentrated HCl was added to it and then covered with a watch glass. The beaker was then placed on a hot plate for 15 minutes to heat. The digestate from the heated sample was filtered using a Whatman No. 41 filter paper into a 100 ml volumetric flask. The digestate was later diluted with a volume of 100 ml and then analysed using a Microwave Atomic Absorption Spectrometer. Concentration levels of the nine different heavy metals elements within the samples were then measured.

### 2.4 Descriptive statistics

The detailed statistical description of all the samples is vital in determination of the basic information about the collected samples. The details are summarized in Table 3. The respective mean can be compared with the recommended limits.

**Table 3:** Summary of the statistical analyses of the data.

Statistic	Arsenic	Mercury	Cadmium	Cobalt	Chromium	Nickel	Lead	Vanadium	Zinc
Mean	0.29055	0.11557	0.59453	0.02716	0.22477	0.29581	0.46230	0.24308	0.53539
standard error	0.07202	0.02166	0.12288	0.00291	0.06872	0.02015	0.12320	0.06089	0.14936
Median	0.13675	0.09705	0.41695	0.02615	0.1005	0.29835	0.214	0.1559	0.38255
standard deviation	0.32211	0.09690	0.54958	0.01304	0.30733	0.09014	0.59089	0.27233	0.66797
Variance	0.10375	0.00938	0.30203	0.00017	0.09445	0.00812	0.34915	0.07416	0.44618
Kurtosis	0.64347	1.90957	−0.05402	−0.43020	3.36933	0.73829	1.67786	4.83650	5.01467
Skewness	1.24345	1.41702	0.93008	0.11240	1.95493	0.61831	1.60778	2.31018	2.28915
Range	1.1011	0.3763	1.8824	0.0481	1.1396	0.3755	1.918	0.9996	2.5263
Minimum	0.002	0.0016	0.0143	0.0039	0.0016	0.1454	0.012	0.0145	0.0263
Maximum	1.1031	0.3779	1.8967	0.052	1.1412	0.5209	1.93	1.0141	2.5526
Sum	5.811	2.3114	11.8906	0.5433	4.4955	5.9162	10.6331	4.8616	10.7079

### 2.5 Correlation coefficient

Numerical value of the correlation coefficient determines the degree of strength and nature of relationship between the observed variables. The result of the Pearson correlation coefficient, Kendall's tau and Spearman rho correlation coefficient are shown in Tables 4a, 4b and 4c.

**Table 4a:** A correlation matrix of the concentration of the heavy metals (Pearson correlation coefficient).

Variables	As	Hg	Cd	Co	Cr	Ni	Pb	V	Zn
As	1								
Hg	0.085	1							
Cd	0.723	−0.382	1						
Co	−0.249	0.578	0.056	1					
Cr	0.671	−0.202	0.817	−0.083	1				
Ni	0.549	0.070	0.152	−0.184	0.018	1			
Pb	0.602	0.160	0.556	−0.319	0.321	0.372	1		
V	0.779	−0.075	0.768	−0.018	0.888	0.212	0.266	1	
Zn	0.283	−0.231	0.302	0.181	0.092	0.504	0.542	0.092	1

**Table 4b:** A correlation matrix of the concentration of the heavy metals (Kendall tau correlation coefficient).

Variables	As	Hg	Cd	Co	Cr	Ni	Pb	V	Zn
As	1								
Hg	0.042	1							
Cd	0.400	−0.347	1						
Co	−0.290	−0.301	0.058	1					
Cr	0.568	−0.179	0.642	−0.047	1				
Ni	0.168	0.116	−0.116	−0.132	−0.032	1			
Pb	0.568	0.011	0.516	−0.280	0.558	−0.011	1		
V	0.579	−0.063	0.379	−0.079	0.547	0.189	0.316	1	
Zn	0.221	−0.253	0.316	0.280	0.337	0.211	0.105	0.347	1

**Table 4c:** A correlation matrix of the concentration of the heavy metals (Spearman rho correlation coefficient).

Variables	As	Hg	Cd	Co	Cr	Ni	Pb	V	Zn
As	1								
Hg	0.116	1							
Cd	0.540	−0.450	1						
Co	−0.410	−0.431	0.205	1					
Cr	0.738	−0.263	0.824	−0.076	1				
Ni	0.292	0.164	−0.111	−0.205	−0.056	1			
Pb	0.774	0.021	0.657	−0.424	0.744	−0.003	1		
V	0.755	−0.113	0.529	−0.110	0.690	0.259	0.484	1	
Zn	0.293	−0.367	0.460	0.397	0.435	0.289	0.189	0.489	1

The implications of the nature and strength of the relationships among the variables are almost consistent among the three different methods.

### 2.6 Analysis of variance

Analysis of variance (ANOVA) was conducted and the result was displayed in Table 5. The result showed that there are significant differences in the means of the heavy metal concentrations of the 20 samples collected from the Ewekoro limestone Formation.

**Table 5:** Analysis of variance (ANOVA) for the samples.

Source of variation	D.F	S.S	M.S	*F*-value	*P*-value
Sample	8	5.4479	0.6801	4.6575	0.00004
Error	171	25.0028	0.1462		
Total	59	52570			
